# Gas Leak Detection by Dilution of Atmospheric Oxygen

**DOI:** 10.3390/s17122804

**Published:** 2017-12-05

**Authors:** Armin Lambrecht, Eric Maier, Hans-Fridtjof Pernau, Thomas Strahl, Johannes Herbst

**Affiliations:** Fraunhofer Institute for Physical Measurement Techniques IPM, Heidenhofstr. 8, D-79110 Freiburg, Germany; Eric.Maier@ipm.fraunhofer.de (E.M.); Hans-Fridtjof.Pernau@ipm.fraunhofer.de (H.-F.P.); Thomas.Strahl@ipm.fraunhofer.de (T.S.); Johannes.Herbst@ipm.fraunhofer.de (J.H.)

**Keywords:** gas sensor, leak detection, tunable laser spectroscopy, infrared absorption, oxygen, nitrogen, methane

## Abstract

Gas leak detection is an important issue in infrastructure monitoring and industrial production. In this context, infrared (IR) absorption spectroscopy is a major measurement method. It can be applied in an extractive or remote detection scheme. Tunable laser spectroscopy (TLS) instruments are able to detect CH_4_ leaks with column densities below 10 ppm·m from a distance of 30 m in less than a second. However, leak detection of non-IR absorbing gases such as N_2_ is not possible in this manner. Due to the fact that any leaking gas displaces or dilutes the surrounding background gas, an indirect detection is still possible. It is shown by sensitive TLS measurements of the ambient background concentration of O_2_ that N_2_ leaks can be localized with extractive and standoff methods for distances below 1 m. Minimum leak rates of 0.1 mbar·L/s were determined. Flow simulations confirm that the leakage gas typically effuses in a narrow jet. The sensitivity is mainly determined by ambient flow conditions. Compared to TLS detection of CH_4_ at 1651 nm, the indirect method using O_2_ at 761 nm is experimentally found to be less sensitive by a factor of 100. However, the well-established TLS of O_2_ may become a universal tool for rapid leakage screening of vessels that contain unknown or inexpensive gases, such as N_2_.

## 1. Introduction

Gas leak detection is an important issue in production processes to prevent safety hazards and to ensure functionality of many products. For natural gas pipeline surveillance, many instrument developments aim at fast and sensitive remote detection of gas leaks. For these purposes gas cameras using modified thermal imaging systems, handheld tunable laser spectroscopy (TLS) instruments, and even helicopter-based systems are available [1,2,3,4,5]. Commercially available handheld TLS instruments in the NIR are able to detect CH_4_ leaks with column densities >1 ppm·m via remote detection. They can be used from a distance up to 30 m and have measurement rates up to 10 Hz [3]. Determination of leak rates is difficult for remote gas detectors since sufficient information about the gas dispersion in the surrounding environment is usually not available. Recently, a TLS remote detection device was able to identify a CH_4_ leak flux of 15 mL/min from a distance of 37 m [6]. However, all these systems rely on infrared absorption of the target gas, which is the case for hydrocarbons, for example. Consequently, leak detection of non-infrared absorbing gases such as N_2_, H_2_, Ar, etc., is not possible in this fashion.

Sensitive leak testing of vessels, tubes, vacuum parts, refrigeration systems, etc., is usually performed via helium (He) leak testing. This detection method is based on a mass spectrometer (MS) that is tuned to He. In most cases, the examined object has to be filled with a He-containing gas. In such a leakage scenario, the He is detectable by pumping (“sniffing”) the leakage gas into the MS through a handheld nozzle located close to the leak. Very small leaks (below 10^−7^ mbar·L/s) can be detected by this method, but a close contact to the object is required. Apart from that, He leak testing from a distance is not possible. Further disadvantages are that the testing is a slow and manual task. A less expensive alternative to He is hydrogen or forming gas that is used in combination with sensitive electrochemical, metal oxide-, or Pd-based detectors. However, the use of hydrogen could be a safety hazard.

Acoustic leak detection is another common method. This method is based on the principle that the leakage gas generates a sound wave which can be detected by a sensitive microphone. Therefore, the object needs to be pressurizing in order to obtain a turbulent flow at the orifice which is required to generate (ultra-) sound waves [7]. This may be circumvented by using an active ultrasound source within the object, but discrimination of the different sound paths could be difficult.

The classical leak detection technique for rather coarse leaks is the well-known “air bubble” method. A vessel under investigation is pressurized with gas (e.g., air) and immersed into a liquid (e.g., water). Air bubbles originating from the leak indicate the leak location and leak size. Minimum leak rates ≥10^−3^ mbar·L/s can be detected by this method which is comparable to acoustic leak detection [8]. Obviously, this method is not practical or feasible for large objects. Furthermore, the method is, generally, a slow and manual task. However, this concept may also be transferred in the gas phase. Therefore, a background gas in the atmosphere substitutes the liquid such that the background gas is displaced and diluted by the leakage gas. Thus, remote leak detection of any target gas—also of non-IR-active gases—should be feasible by a sensitive tunable laser spectroscopy (TLS) measurement of the background gas concentration. Ubiquitous and IR-active background gases are, for instance, CO_2_, O_2_, or H_2_O, which should be different from the target gas. In order to detect a leak-induced ambient background concentration change, the distance has to be fixed or measured simultaneously [9]. This can be achieved with state-of-the-art laser rangefinders.

In the next section, we show that O_2_ is the optimum candidate for a suitable background gas. Then the experimental setup is explained. Because of the visibility of the employed laser we use the term “LeakEye” for our technique. Computational fluid dynamics (CFD) simulations of the experimental setup are performed using COMSOL Multiphysics® (COMSOL Inc., Burlington, MA, USA). Then the results of experiments on extractive detection—later on, standoff detection—are given. In this work the terms non-tactile and standoff detection are synonymously used, even for rather short distances. The essential difference to extractive detection is that gas flow is not disturbed. We shall always compare experiments with direct (positive) detection of CH_4_ and with corresponding experiments on indirect (negative) detection of O_2_.

## 2. Modelling

The fundamental relation of absorption spectroscopy is given by the Beer-Lambert law:

$$I(\upsilon )=I_{0}\cdot e^{-\alpha (\upsilon )\cdot d\cdot c}\;\mathrm{with}\;\alpha (\upsilon )=S(T,\upsilon_{0})\cdot g(p,T,\upsilon -\upsilon_{0})$$

where *I*(υ) is the frequency-dependent transmitted intensity after passing a measurement cell with an optical path length *d*, *c* is the gas concentration, and *α*(*υ*) is the frequency-dependent gas specific absorption coefficient, which is given by the product of the temperature-dependent line strength *S*(*T*,υ_0_) at the characteristic absorption frequency υ_0_, and the pressure- and temperature-dependent line shape function *g*(*p*,*T*,υ − υ_0_).

As a rule of thumb we assume that a 1% reduction of the effective absorption length *d* by dilution of the background gas by the leakage gas has to be measured with a laser spectroscopic setup. In a typical situation an undisturbed extension of the leaking gas plume up to approximately 1 cm from the surface of the leaking object is assumed. For larger distances strong dilution by ambient air flow is expected. As a result the “LeakEye” technique is practically limited to standoff distances of approximately 1 m. For larger distances the relative measurement effect is too small.

Relevant atmospheric background gases, such as O_2_, H_2_O, and CO_2_, with infrared absorption data are available in the HITRAN database [10]. Suitable absorption lines of these gases for typical ambient concentrations, *T* = 296 K and *p* = 1013 mbar were selected and laser spectroscopic measurements were simulated using Mathcad (PTC, Unterschleißheim, Germany). For a total optical distance of 200 cm the transmission signal change for a 1% change of the optical distance was determined. For all gases distributed feedback (DFB) laser diodes in the near infrared (NIR) are available. In the simulation laser emission powers were set to 3 mW. Photodiodes with optimum detectivities with respect to noise equivalent powers (NEP) for the selected wavelength ranges were chosen as detectors. A maximum total transmission of 1% was assumed to account for the weak signal for standoff detection. The resulting power changes at the detector ΔP with respect to the relative power changes ΔP_rel_ are shown in Table 1. 

The measurement of O_2_ at 761 nm was selected as the best choice (cf. Table 1): although TLS-based hygrometers could serve as an established platform [11], the expected ambient fluctuations for H_2_O, as well as for CO_2_, are too high. The first experiments with CO_2_ show that the measurements are strongly affected by the exhaled air of people in the lab; this effect is much stronger for CO_2_ than for O_2_. Additionally, for CO_2_ at 2004 nm the sensitivity is expected to be limited by detector noise. A further reason for the choice of O_2_ with respect to for the measurement at 761 nm is that TLS of O_2_ has become an established industrial measurement technique. For commercially-available process instrumentation, a precision of 10^−3^ for O_2_ concentration measurements is achieved [12,13], and laser radiation at 761 nm is still visible, which is a significant advantage for the adjustment of experimental setups.

A further result of the simulation is that for a standoff distance of 1 m a direct measurement of a 1 cm long CH_4_ gas column at 1651 nm is expected to be approximately more sensitive by a factor of 1000 than an indirect O_2_ measurement by the displacement of 1 cm background air. Therefore, for a better comparison between both methods we use 1% CH_4_ in N_2_ for direct detection.

## 3. Experimental Details

Gas detection by tunable diode laser spectroscopy is an established technique. We are employing the direct spectroscopy scheme. With this method, the measured absorbance spectra are fitted with calculated gas absorption lines using HITRAN line parameters. Knowing the temperature, pressure, and the absorption length, the gas concentration is obtained [14].

For the measurements a test leak was made. This consists of a shot-blasted 100 × 100 mm^2^ Al-plate with a central 1 mm diameter orifice. The blasted surface ensures sufficient diffuse reflection of incident radiation. Leakage gas is ejected from the orifice at different flow rates between 1000 mL/min and 1 mL/min. A gas supply hose is connected at the back side of the plate. Flow rates and the leakage gas composition are set using a HovaCAL digital 922 SP flow controller (IAS GmbH, Oberursel, Germany).

**Figure 1:**
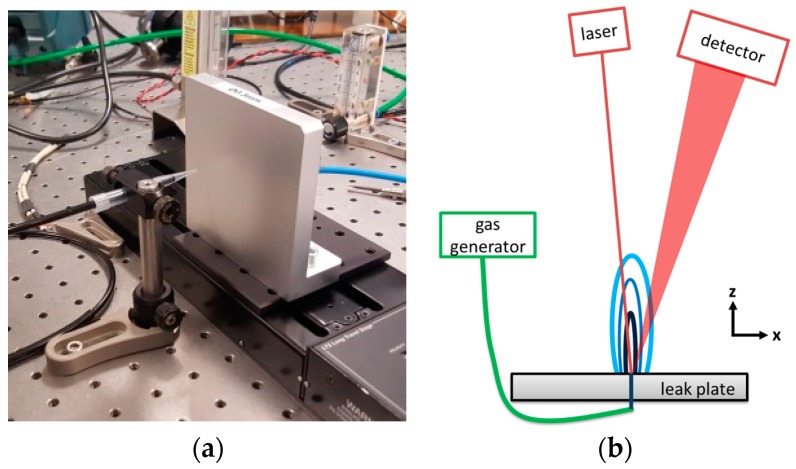
(**a**) Leak plate mounted on a linear displacement stage. The tip of an extractive sampling probe is positioned at 10 mm distance in front of the plate; and (**b**) the schematic setup for standoff leak detection.

The leak plate (Figure 1) is mounted on a sliding stage to displace the leak position horizontally on the table. For extractive measurements, the gas is sampled by a hose with a 1 mm sampling tip mounted in a distance of 10 mm from the leak plate. The gas is pumped through a measurement cell with an optical path length of 84 cm and a volume of 175 mL. We used pump rates of a diaphragm pump between 100 mL/min and 1000 mL/min. Pressure fluctuations are minimized by an additional buffer volume between the pump and measurement cell.

For standoff measurements the laser and detector are positioned at a distance from the leak plate. The diffuse reflection of the collimated laser beam is collected by an f = 11 cm, 2” focusing lens in front of a Si-photodiode. Typically, distances of 55 cm and 52 cm of the laser with respect to the detector lens from the leak plate are used. The angle between the laser and detector beams is approximately 17°.

For static measurement, the leakage flow rates are varied in steps and the leak position is fixed at a maximum signal for extractive or standoff detection. For dynamic measurements, the leak plate is moved horizontally back and forth with a speed between 0.2 mm/s and 5 mm/s for a fixed leak flow rate, which is changed after a couple of scans.

Positive or direct detection is performed with 1% CH_4_ in N_2_. For negative or indirect detection we used 100% N_2_. For the CH_4_ measurements, a 1651 nm pig-tailed single-mode laser diode in a butterfly mount was employed (Eblana Photonics Ltd., Dublin, Ireland). For detection, an InGaAs-photodiode type G 12182-030 (Hamamatsu Photonics GmbH, Herrsching, Germany) with a 3 mm diameter was used. For the O_2_ measurements we used a C-mount-DFB laser diode emitting at 761 nm (nanoplus GmbH, Gerbrunn, Germany) in a custom laser housing and a quadratic 3.6 mm × 3.6 mm Si-photodiode. The lasers were driven with a standard benchtop laser current driver/temperature controller (ILX Lightwave LDC 3722, Newport Corporation, Irvine, CA, USA). Laser control, data acquisition, and evaluation were performed with a proprietary electronics board and LabVIEW (National Instruments Corporation, Austin, TX, USA).

## 4. Experimental Results

### 4.1. Extractive Measurements with O_2_

**Figure 2:**
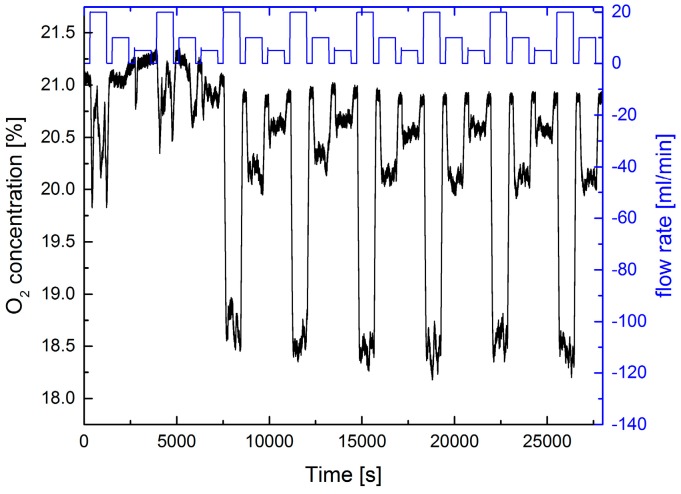
Extractive static measurement of the O_2_ concentration using a 0.5 mm diameter tip in a distance of 10 mm from the leak plate. A pump rate of 100 mL/min was employed. The upper blue curve shows the variation of the N_2_-flux from the leak plate vs. time (right ordinate axis). Flux steps of 20, 10, and 5 mL/min were used.

Using the setup described above we investigated the extractive detection of a N_2_ flow from the leak plate using the indirect O_2_ detection method. The result of a static measurement is shown in Figure 2.

At the beginning of the measurement we had to adjust the position of the sampling tip in front of the leak plate. The whole setup was mounted on an optics table in the lab (see Figure 1). On this table other experiments were performed simultaneously, and staff was nearby moving around in the lab. Obviously they are disturbing the experiment. This activity ceased after 18:00 h and the experiment was running automatically over night for approx. another 6 h. After approximately 2 h fewer disturbances by fluctuations of the gas flow distribution (and of the temperature in the lab) occur, distinct N_2_ flow steps are visible, and flow rates of 5 mL/min can be clearly observed. With the applied pump rate of 100 mL/min it takes nearly 2 min to exchange the gas in the cell. Fluctuations in the N_2_ gas flow during this time have to be avoided. The observed signal-to-noise-ratio (SNR) indicates that steps of 2 mL/min would be resolvable. Thus, leak rates below 0.1 mbar·L/s could be detected by this method. Due to the long duration of the individual flow steps in Figure 2 a complete gas exchange in the measuring cell is ensured for a pump rate of 100 mL/min.

**Figure 3:**
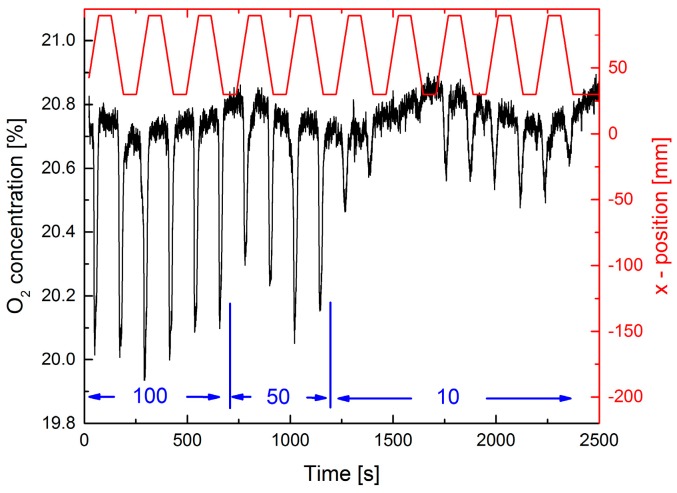
Extractive dynamic measurement of the O_2_ concentration using a 0.5 mm diameter tip at a distance of 10 mm from the leak plate. A pump rate of 500 mL/min is used. The upper red curve shows the position of the sliding stage which moves back and forth with 1 mm/s. The leak position is at 60 mm (right ordinate scale). Three different N_2_-fluxes of 100, 50, and 10 mL/min are applied as indicated in blue.

In Figure 3 a dynamic measurement is displayed. A scan speed of 1 mm/s for the sliding stage was chosen, which seems quite realistic for manual leak searching. In contrast to the static measurements the O_2_-concentration dips at the leak position show considerable fluctuations. For 10 mL/min some of the dips are completely missing. This has two reasons: First, the speed of the sliding stage is too fast for the extractive technique even at a pump rate of 500 mL/min. Thus, a complete exchange of the gas in the cell is not possible when the leak position is passing by in front of the probe tip. This behavior is an essential disadvantage of any extractive method. The other reason is that, for low rates, the flow is more likely deflected by any small air turbulence in the lab. However, for a repetitive measurement, detection of a leak with 10 mL/min, i.e., with a leak rate above 0.1 mbar·L/s, is feasible.

### 4.2. Extractive Measurements with CH_4_

**Figure 4:**
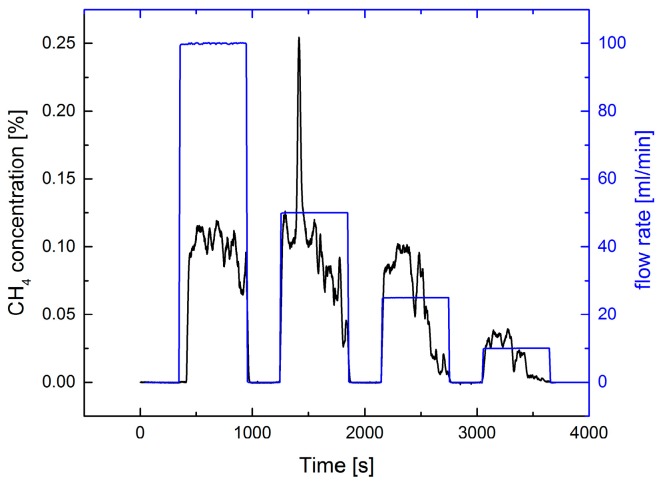
Extractive static measurement of the CH_4_ concentration using a 0.5 mm diameter tip at a distance of 10 mm from the leak plate. A pump rate of 100 mL/min was employed. The blue curve shows the variation of the gas flux from the leak plate vs. time (right ordinate axis). Flux steps of 100, 50, 25, and 10 mL/min were used.

**Figure 5:**
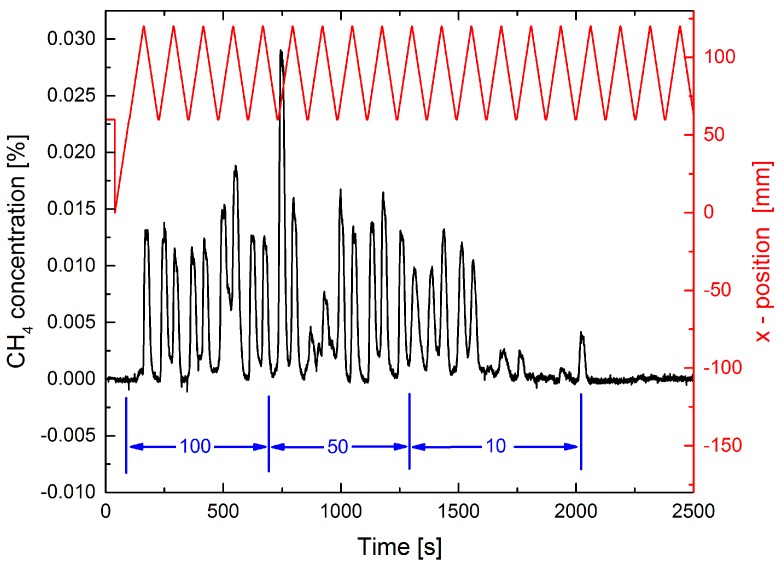
Extractive dynamic measurement of the CH_4_ concentration using a 0.5 mm diameter tip at a distance of 10 mm from the leak plate. A pump rate of 100 mL/min was employed. The upper red curve shows the position of the sliding stage, which moves ±30 mm away from the leak position at 90 mm (right ordinate scale). The speed is 1 mm/s. Three different N_2_-fluxes of 100, 50, and 10 mL/min are applied, as indicated in blue.

For comparison measurements, using the positive detection scheme with 1% CH_4_ in N_2_ was performed with an identical setup (see Figure 4 and Figure 5).

In the case of the static measurement (Figure 4) strong concentration fluctuations were observed, but a 10 mL/min leak flux could be clearly detected. The observed concentration values do not scale with the employed gas fluxes, probably because of the gas flow distribution (in Figure 2 smaller fluxes were used). The noise equivalent concentration (1 σ, 60 s average) of ≈1.3 ppm for zero gas flux is much lower than the observed concentration values of ≈300 ppm at a gas flux of 10 mL/min. Due to the higher absorption line strength of CH_4_ the SNR is higher than for O_2_. Leak detection is limited by the fluctuations. We further observed that increasing the pump rate does not help to reduce these fluctuations and concentration values are smaller due to dilution.

The dynamic CH_4_ measurement in Figure 5 is qualitatively similar to the results displayed in Figure 3. Due to the lower pump rate the gas exchange time in the measurement cell is longer than in Figure 3. Thus, strong fluctuations and missing peaks are observed. The concentration values are much smaller than in Figure 4 because the cell is only partly filled when the leak position passes the tip. For repetitive measurements detection of fluxes of 10 mL/min is feasible, but lower fluxes are hardly observable despite the high SNR. However, for 100% CH_4_, this result may be scaled to detectable leak rates in the range of 10^−3^ mbar·L/s.

### 4.3. Standoff Measurements with O_2_

For standoff detection the setup of Figure 1b was used. To ensure the sensitivity of the method, first the distance between laser and leak plate was varied without any additional N_2_ flux.

**Figure 6:**
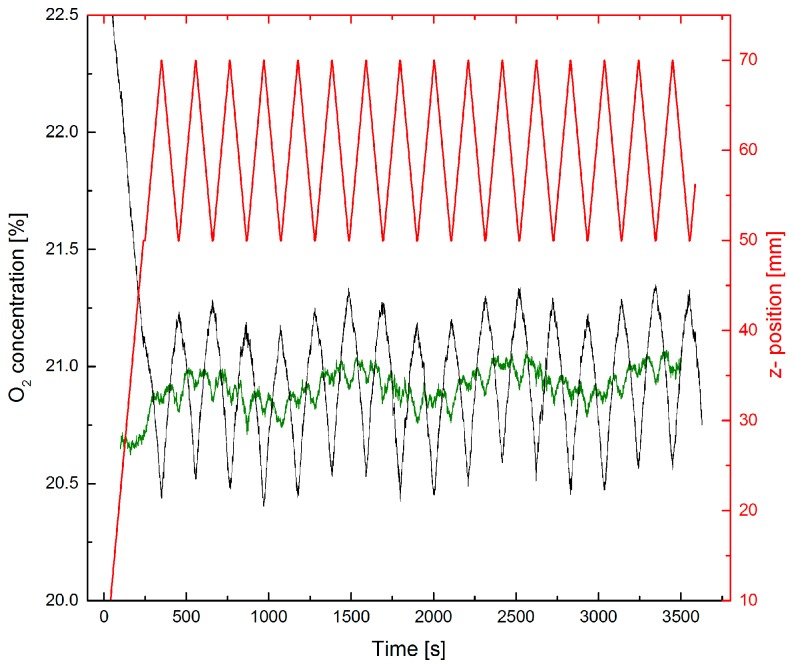
Distance variations during standoff O_2_ measurements in ambient air using the leak plate as diffuse reflector. The leak plate was moved back and forth towards laser and detector for ± 1 cm (red curve, right ordinate scale). The resulting change of the O_2_ concentration assuming a constant optical distance of 114 cm (laser-plate-detector) is shown. The green curve results from a later correction of the concentration measurement by the factor 114 cm/actual distance.

The observed relative change of the O_2_ concentration in Figure 6 corresponds to the relative change of the optical path length. The small noise of the concentration signal in Figure 6 indicates that changes of the path length or the O_2_-concentration below 1% could be detected. Correction for the distance changes in Figure 6 leaves a slow variation of the O_2_ concentration with a period of ≈15 min. This is an artifact caused by temperature fluctuations of the laboratory air conditioning system influencing our TLS electronics and not a systematic concentration fluctuation of the lab air.

**Figure 7:**
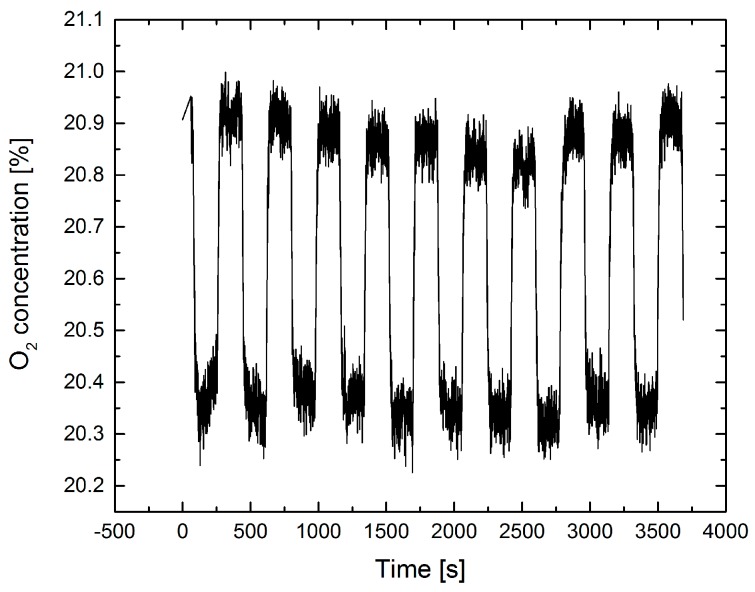
Standoff O_2_ measurement using a diffuse reflecting gas cell instead of the leak plate of Figure 1b. The total optical path length (laser-diffuse reflecting backplate-detector) was 114 cm. The optical path length in the cell was 30 mm. The gas in the cell was periodically changed from lab air to 100% N_2_.

A further confirmation of the feasibility of the indirect method is obtained by the following experiment: a gas cell with 15 mm length and a diffuse reflecting backplate (DG10-120, Thorlabs Inc., Newton, NJ, USA) was inserted into the laser beam at the position of the leak plate in Figure 1b). Due to the small cell volume of ≈6.5 mL a rapid gas exchange is possible. Figure 7 shows the result of an experiment exchanging the air in the cell by N_2_. The observed reduction in total average O_2_ concentration for an optical path length of 114 cm agrees very well with the calculated of Δc = 20.9% × (3 cm/114 cm) = 0.55%.

**Figure 8:**
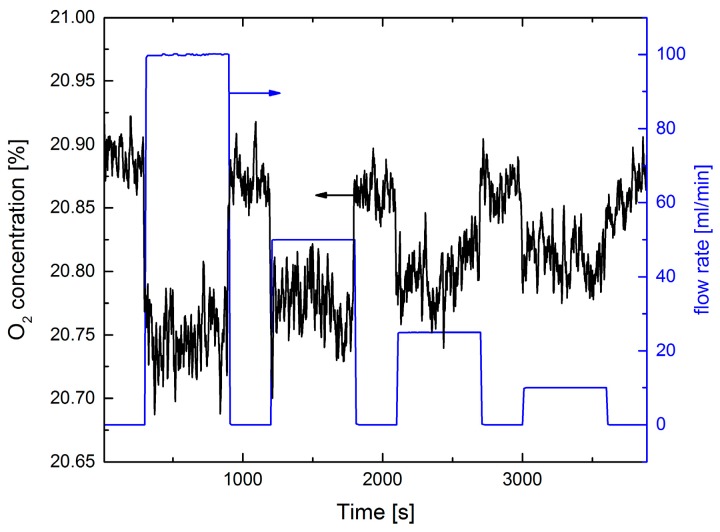
Static standoff N_2_ detection via an O_2_ measurement. The distance between laser and leak plate was 55 cm. The N_2_ flux was stepwise reduced from 100, 50, 25, to 10 mL/min (blue curve, right ordinate scale).

Directing the collimated laser spot (diameter 2–3 mm) onto the gas orifice on the leak plate with defined N_2_ fluxes, a static leak test is performed (Figure 8). Fluxes of 10 mL/min can be detected with a SNR ≈ 2, corresponding to an O_2_ concentration resolution of ≈200 ppm in this experiment. However, the observed concentration steps do not linearly follow the N_2_ fluxes: a ten-fold increase of the N_2_ flux yields only a three times stronger concentration dip. This indicates that only a small part of the effusing N_2_ gas jet from the leak is intersecting with the laser radiation for higher fluxes. CFD simulations support this argument (see Section 4.6).

**Figure 9:**
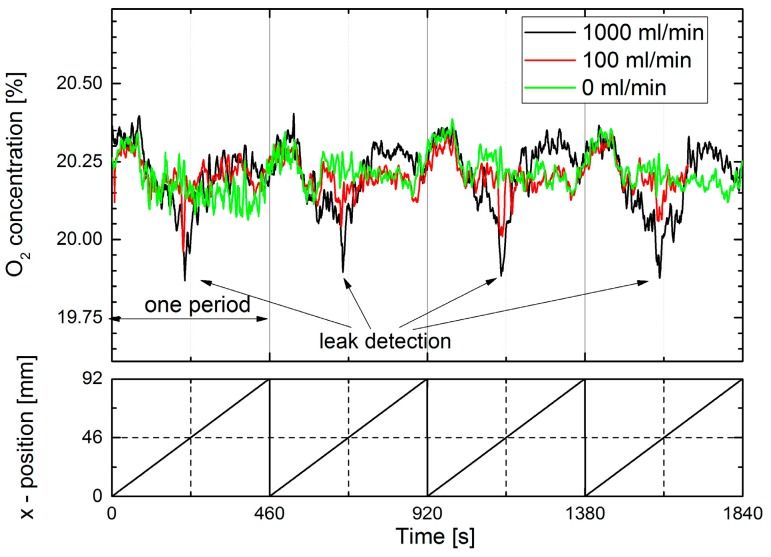
Standoff dynamic N_2_ leak detection via measurement of the O_2_ concentration. The distance between the laser and leak plate was 55 cm. The leak plate was horizontally moved by a sliding stage with a speed of 0.2 mm/s in a sawtooth manner, as indicated by the lower figure. The leak position is at 46 mm.

Results of a dynamic standoff detection experiment are shown in Figure 9. Similar to the extractive measurements in Figure 3, the leak plate was moved perpendicular to the interrogating laser beam.

N_2_ fluxes of 100 mL/min could be dynamically detected and localization of the leak is feasible. However, the dynamic detection is significantly less sensitive than the static detection in Figure 8. Furthermore, a fixed pattern noise was observed by moving the laser spot across the diffuse reflecting plate. Increasing the laser spot size by a factor of three did not improve the result.

### 4.4. Standoff Measurements with CH_4_

**Figure 10:**
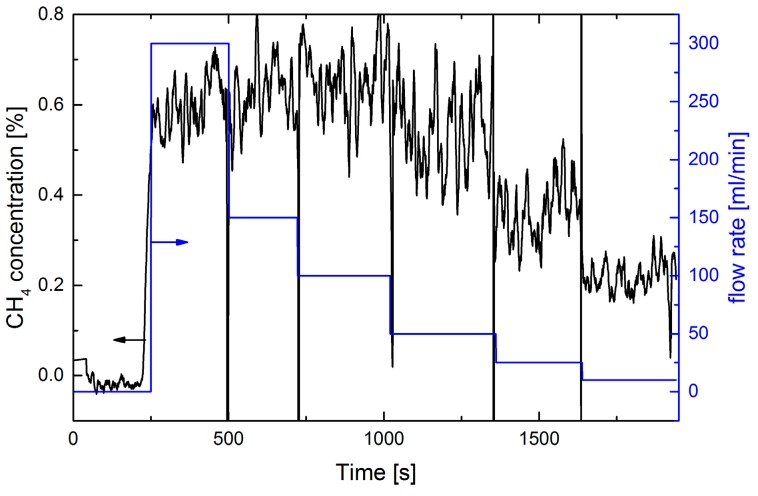
Static standoff measurement of 1% CH_4_ in N_2_. The distance between laser and leak plate was 55 cm. The flux was stepwise reduced from 300, 150, 100, 50, 25, to 10 mL/min (blue curve, right ordinate scale).

As with the extractive technique measurements with 1% CH_4_ in N_2_ were performed with an identical setup. For the static measurement a similar nonlinear behavior was found (see Figure 10). This supports the assumption that the leakage gas jet is only partly intersected by the laser radiation, and a saturation of the signal occurs at high fluxes. The low noise level for zero flux indicates that even lower flux rates than 10 mL/min should be detectable by the static method.

**Figure 11:**
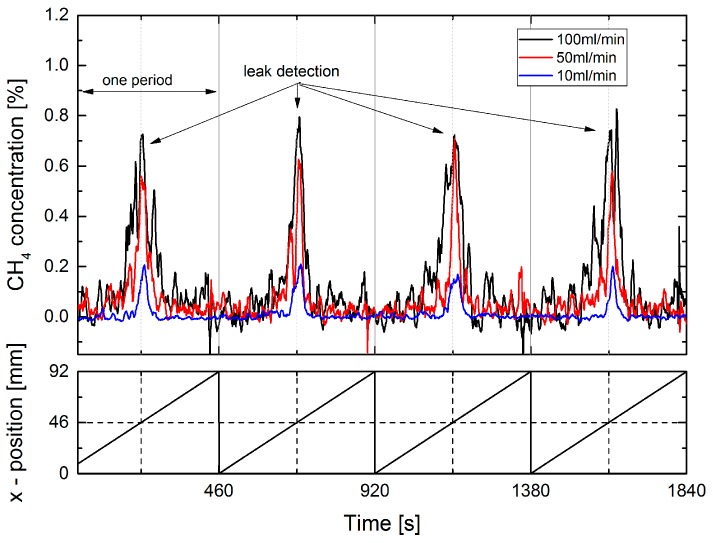
Dynamic standoff measurement of 1% CH_4_ in N_2_ with fluxes of 100, 50, and 10 mL/min. The distance between laser and leak plate was 55 cm. The leak plate was horizontally moved by a sliding stage with a speed of 0.2 mm/s in a sawtooth manner, as indicated by the lower figure. The leak position is at 46 mm

A dynamic standoff measurement with CH_4_ is shown in Figure 11. In contrast to the indirect detection scheme a leak flux of 10 mL/min could be dynamically detected and localized. The low noise between the leak locations indicates that even lower fluxes should be detectable by this method.

### 4.5. Further Experiments

The leak plate setup was also adapted for measurements with a commercial helium leak tester (SmartTest HLT 560, Pfeiffer Vacuum GmbH, Asslar, Germany) in the sniffing mode. For this purpose the nozzle of the sniffing probe was mounted at the position of the extractive tip in Figure 1a. Dynamic measurements similar to those of Section 4.1 and Section 4.2 were performed with 10% He in N_2_. Despite the superior sensitivity and dynamic range of the helium leak tester, localization of a 10 mL/min flow from the leak plate was more difficult than expected. In order to prevent a rapid increase of the He-background concentration, the experiments were performed under a fume cupboard. The nozzle of the sniffing probe had to be at a closer distance from the leak plate (i.e., 6 mm compared to 10 mm in Figure 1a). Compared to CH_4_ or N_2_, He is diffusing sideways more rapidly from the orifice, which tends to make leak localization more difficult than with the other techniques.

### 4.6. CFD Simulations

**Figure 12:**
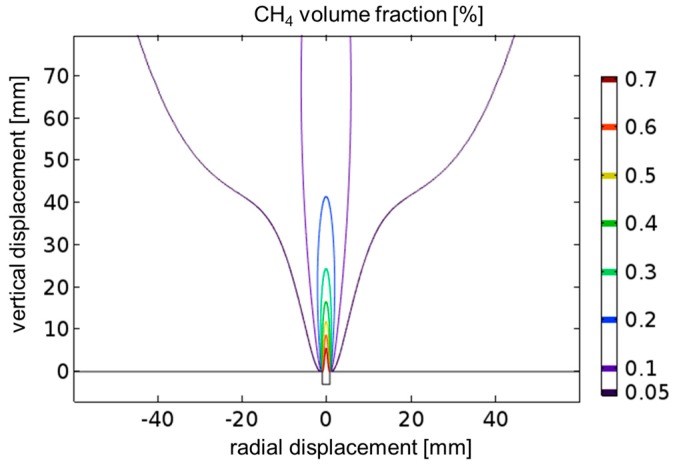
CFD simulation using COMSOL for a flux of 1% CH_4_ in N_2_ effusing from an orifice of 1 mm diameter with 100 mL/min into ambient air. An axial cut along the gas flow direction through the centrosymmetric laminar flow distribution shows iso-concentration lines of the CH_4_ volume fraction.

Obviously the sensitivity of the measurements is limited by the gas flow distribution and its fluctuations. At the open atmosphere influence of wind and soil properties around a leak will be severe. Even in the lab, air conditioning, ventilators of electronic devices, and the presence of lab personnel result in local air turbulence which affects the measurements. For a better understanding of the gas flow distribution for the leak plate (see Figure 1) CFD simulations using COMSOL (turbulent multiphase flow) are performed (Figure 12). For a flow of 100 mL/min the gas is ejected in a narrow laminar flow jet. The simulation model utilizes the rotational symmetry of the setup so the simulation is effectively done in 2D and rotated around the center axis. The model was designed similar to the COMSOL combustion model [15], but without any reaction. The simulated volume above the orifice in Figure 12 has a diameter of 12.5 cm and a height of 17.8 cm. A radial influx of air (80% N_2_ and 20% O_2_) is allowed in the model. As in the laboratory experiment no influx (tight wall) is allowed from the region with a vertical displacement smaller than 0 mm (indicated by the solid black line within Figure 12). All other boundary conditions of the simulated volume to the universe are set to “no pressure drop”. The surrounding pressure is set to 1 bar. To validate the simulation results a two times larger diameter and height has also been used, but the results did not differ from the results shown in Figure 12. The temperature of all components is set to 20 °C.

The CH_4_ concentration is reduced right at the orifice by dilution from air from the side, similar to a jet pump. Within the jet the maximum concentration decreases rapidly and is lower than 0.4% at a 20 mm distance. The full width at half maximum at a 20 mm distance from the leak plate is less than 10 mm. These findings were experimentally confirmed by scanning the gas jet with a commercial extractive instrument (IRwin™, Inficon GmbH, Cologne, Germany).

When the gas flux is increased from 25 mL/min to 200 mL/min the jet gets longer, but the shape is almost unchanged. Variation of the flux parameter within the CFD simulation, as well as thermal imaging experiments with CO_2_ jets, show, qualitatively, a similar behavior. For higher fluxes, turbulence sets in. For small fluxes the distribution is deflected by environmental air flow [16].

As already mentioned, the results of standoff measurements are strongly affected by the intersection of the laser beams and the leak flux distribution. Thus, only qualitative results can be obtained.

## 5. Discussion

In this work, we have shown that gas leaks can be detected by the dilution of ambient O_2_. The fact that the leakage gas reduces the O_2_ concentration in the vicinity of the leak can be measured. The measurement principle is based on tunable laser spectroscopy, which was realized in an extractive and a standoff device, respectively. The method is demonstrated for N_2_ as the leakage gas, which is not an infrared absorber. The technique can be applied for other non-infrared absorbing gases, such as H_2_ or Ar, as well as for any gas, except for the ambient gas itself. Instead of O_2_, other infrared-absorbing background gases may be used, e.g., CO_2_ or H_2_O. However, CO_2_ and H_2_O concentrations show strong local and temporal fluctuations which have to be compensated. Consequently, an interesting application field could be an environment with a controlled atmosphere (e.g., in a greenhouse).

For static extractive detection N_2_ fluxes of less than 5 mL/min can be observed, corresponding to a detectable leak rate L ≥ 0.1 mbar·L/s. In consideration of a limited response speed, comparable results are obtained in a dynamical extractive detection scheme. Almost the same sensitivity level can be achieved in the case of static standoff detection from a distance of about 0.6 m. In a dynamic standoff detection scenario, only flows of 100 mL/min could be detected. The localization of the gas leak is feasible within ±5 mm. Obviously, this value depends on system parameters, such as the speed of displacement (leak), pump rate (extractive detection), or the laser spot size (standoff detection).

Comparing the direct measurements using 1% CH_4_ in N_2_ and the indirect measurements using dilution of the ambient 21% O_2_ by N_2_, the obvious result is that the sensitivity for the direct method is higher. On the one hand, the difference in the absorption line strength between CH_4_ at 1651 nm (10^−21^ cm^−1^·mol^−1^·cm^2^) and O_2_ at 761 nm (8 × 10^−24^ bcm^−1^·mol^−1^·cm^2^) is about two orders in magnitude [10]. On the other hand, Si-photodiodes used for O_2_ have a ten-fold higher detectivity than the InGaAs diodes needed for CH_4_. This helps to reduce the effect of the large line strength difference. Our experiments indicate almost the same SNR for both methods since the influence of gas fluctuations is dominating.

For 1% CH_4_ in N_2_ the fluctuations of the signal with leak flux are larger than without flux (cf. Figure 10). This effect is hardly observable for O_2_ since the ambient concentration shows a higher noise level. Consequently, the detection is practically limited by the fluctuations caused by gas flow instabilities. Therefore, the estimation of leak rates L ≥ 10^−3^ mbar·L/s for a 100% CH_4_ standoff measurement seems to be quite optimistic. Looking at these fluctuations could be another promising method of leak detection that needs to be investigated in further experiments.

Furthermore, it is shown that the extractive method is more sensitive than the standoff one. In addition, the extractive method can be improved by optimization of the gas cell and pump rate. For O_2_, a compact Herriott gas cell with an optical path length of 5 m was reported in [17]. The sensitivity level of the above mentioned ultrasound method may be achieved by using such cell designs and leak rates <10^−2^ mbar·L/s should be detectable. Even higher path lengths can be obtained by using resonant cells and corresponding detection schemes [18,19], which are established for high-resolution gas detection. However, for this application small changes relative to a high ambient background have to be detected. Thus, special differential cell designs or frequent fast gas exchange with ambient air as a reference gas will be required to increase the sensitivity. For the short time intervals typical for leak detection, a relative sensitivity in the O_2_ concentration below 10^−4^ may be detectable. This corresponds to a concentration change of 20 ppm within a 21% background concentration level.

The sensitivity of standoff techniques strongly depends on the reflection properties of the backscattering surface [20]. A low reflectivity reduces the detected radiation intensity and the SNR. This holds for CH_4_ detection at 1651 nm, as well as for O_2_ detection at 761 nm. Good results are achieved with objects of high diffuse reflectivity, e.g., the reflecting plate shown in Figure 7. The leak plate of Figure 1a has a stronger specular reflection. However, at a scattering angle of 17° the reflectivity is similar to the value of the Thorlabs DG10-120 plate. In real applications non-cooperative specular reflectors are most difficult because they reflect in any direction. Specular reflection of common objects increases with increasing wavelength. Thus, working at 761 nm is advantageous compared to 1651 nm.

Improvement of the standoff technique might be feasible for short distances around 0.2 m to 0.3 m. Depending on the backscattering properties of leaking objects the optical beam diameter and the aperture of the receiving optics may be enlarged. Therefore, the interaction of laser radiation and the leakage gas plume relative to the total absorption path length could be increased such that minimum leak rates ≥10^−2^ mbar·L/s may be detectable.

In conclusion, we have shown the feasibility of gas leak detection by dilution of atmospheric O_2_. This indirect method has several advantages in spite of its lower sensitivity compared to direct detection of infrared absorbing gases. Since the method is independent of the leakage gas, it can be used as a universal leak sensor. For instance, a combination with another specific infrared gas detector might be possible. Compared to the established ultrasound technique, leaks can be detected which do not generate sound. Detection of visible radiation at 761 nm enables the use of fast, highly-sensitive, and inexpensive standard photodiodes. Large area detectors, special sensor geometries and arrays, are commercially available. Imaging leak detection is also feasible by the use of a laser scanning system in combination with fast data processing.

The most important application of TLS in terms of market share represents the measurement of O_2_. In process industries, TLS instruments from several suppliers are commercially available. As a consequence, component prices are generally lower compared to other TLS applications. In the case of O_2_ applications, VCSEL laser diodes can be used, which are less expensive than DFB lasers. Furthermore this laser type can be manufactured in large volumes and small packages. In this context, an integration of a laser-based O_2_ sensor in a mobile phone might be possible. 

## Figures and Tables

**Table 1:** Selection of background gases and detection wavelengths.

Gas	O_2_	H_2_O	CO_2_
Wavelength(nm)	761	1392	2004
Concentration	21% + 1% H_2_O	1%	450 ppm + 1% H_2_O
Detectivity D * (Jones)	10^13^	10^12^	10^11^
NEP (@100 kHz, nW)	0.01	0.08	0.8
ΔP(nW)	12	60	2
ΔP_rel_	4 × 10^−4^	3 × 10^−3^	1.5 × 10^−4^
Suitable	+	− ^1^	− ^1,2^

^1^ ambient concentration fluctuations; ^2^ detector noise limitation.
